# Erratum for Monk et al., Complete Bypass of Restriction Systems for Major *Staphylococcus aureus* Lineages

**DOI:** 10.1128/mBio.00171-16

**Published:** 2016-03-01

**Authors:** Ian R. Monk, Jai J. Tree, Benjamin P. Howden, Timothy P. Stinear, Timothy J. Foster

**Affiliations:** a Moyne Institute of Preventative Medicine, Department of Microbiology, School of Genetics and Microbiology, Trinity College Dublin, Dublin, Ireland; b Department of Microbiology and Immunology, University of Melbourne at the Doherty Institute for Infection and Immunity, Melbourne, Australia; c Austin Centre for Infection Research (ACIR), Infectious Diseases Department, Austin Health, Melbourne, Australia; d Microbiological Diagnostics Unit, University of Melbourne at the Doherty Institute for Infection and Immunity, Melbourne, Australia

## ERRATUM

Volume 6, no. 3, doi:10.1128/mBio.00308-15, 2015. The authors inadvertently omitted the accession numbers for PacBio sequence runs on the *Staphylococcus aureus* (MW2, NRS384, MRSA252, and JKD6159) and *Escherichia coli* (IM01B, IM08B, IM30B, and IM93B) isolates. These accession numbers are available from http://www.ebi.ac.uk/ena under the study accession number PRJEB10032 (see Table 1 below for strain run accession numbers). Additionally, the *E. coli* strains described in the paper have been deposited and are available from BEI Resources (https://www.beiresources.org/) and the Belgium Co-ordinated Collections of Microorganisms (http://bccm.belspo.be/).

**TABLE 1:** PacBio sequence run accession numbers

Run accession no.	Strain sequenced
ERR1213691	MW2
ERR1213692	NRS384
ERR1213693	MRSA252
ERR1213694	JKD6159
ERR1213695	IM01B
ERR1213696	IM08B
ERR1213697	IM30B
ERR1213698	IM93B

